# INSL4 as prognostic marker for proliferation and invasiveness in Non-Small-Cell Lung Cancer

**DOI:** 10.7150/jca.51332

**Published:** 2021-05-05

**Authors:** Damiano Scopetti, Danilo Piobbico, Cinzia Brunacci, Stefania Pieroni, Guido Bellezza, Marilena Castelli, Vienna Ludovini, Francesca Romana Tofanetti, Lucio Cagini, Angelo Sidoni, Efisio Puxeddu, Maria Agnese Della-Fazia, Giuseppe Servillo

**Affiliations:** 1 Department of Medicine and Surgery, Section of General Pathology, University of Perugia, Perugia- Italy.; 2 Department of Medicine and Surgery, Section of Anatomic Pathology and Histology, University of Perugia, Perugia- Italy.; 3 Medical Oncology, S. Maria Della Misericordia Hospital, Perugia, Italy.; 4 Department of Medicine and Surgery, Section of internal medicine, angiology and atherosclerosis diseases.; 5 Department of Medicine and Surgery, Section of internal medicine and endocrine and metabolic sciences.

**Keywords:** INSL4, NSCLC adenocarcinoma, invasiveness, proliferation, xenograft mouse

## Abstract

Non-small-cell-lung cancer accounts for 80-85% of all forms of lung cancer as leading cause of cancer-related death in human. Despite remarkable advances in the diagnosis and therapy of lung cancer, no significant improvements have thus far been achieved in terms of patients' prognosis. Here, we investigated the role of INSL4 - a member of the relaxin-family - in NSCLC. We overexpressed INSL4 in NSCLC cells to analyse *in vitro* the growth rate and the tumourigenic features. We investigated the signalling pathways engaged in INSL4 overexpressing cells and the tumour growth ability by studying the tumour development in a patient derived tumour xenograft mouse model. We found an INSL4 cell growth promoting effect *in vitro* in H1299 cells and *in vivo* in NOD/SCID mice. Surprisingly, in NSCLC-A549 cells, INSL4 overexpression has not similar effect, despite huge basal *INSL4-mRNA* expression respect to H1299. The *INSL4-mRNA* analysis of eight different NSCLC-derived cell lines, revealed highly difference in the *INSL4-mRNA* amount. Transfection of NSCLC lines with INSL4-Myc showed huge level of *INSL4-mRNA* with a very low amount of protein expressed. Notably, similar discrepancy has been observed in NSCLC patients. However, in a cohort of NSCLC patients analysing a database, we found a significant inverse correlation between *INSL4* expression and Overall Survival. By combining the *in vitro* and *in vivo* results, suggest that in patients whose NSCLC adenocarcinoma spontaneously expressed high levels of *INSL4* post-transcriptional modifications affecting INSL4 do not allow to assess precision therapy in selected patients without consider protein INSL4 amount.

## Introduction

Lung cancer (LC) is the leading cause of cancer-related death in human. Non-Small Cell Lung Cancer (NSCLC) adenocarcinoma is responsible for more than 80-85% of the LC, and the expected 5-year survival rate of NSCLC patients is about 15% 1-4. Despite new drugs and therapeutic regimens, the prognosis for LC patients has not significantly changed over the last 20 years. However, knowledge of the molecular biology of LC has greatly improved. Because of such a deeper insight into the multiple somatic mutations and gene expression changes that occur in LC 5-7, appropriate - if not personalized - therapeutic approaches represent an available goal for patients with a defined gene expression profile. Many studies have thus been attempting to identify genes that could be exploited in prediction models for assessing the risk of recurrence based on surgical LC specimens, but the mechanistic roles of those genes still remain unclear 8, 9.

To gain molecular insight into early-stage NSCLC of recently resected patients with stage I adenocarcinoma, we analysed the gene expression profile of Early Relapse (ER) versus No Relapse (NR) NSCLC specimens 10. We identified a number of genes differentially expressed by ER compared to NR NSCLCs. Such association could be established between a specific gene-signature and patient prognosis. In particular, a set of ten genes were identified whose upregulation or downregulation correlated with early recurrence. Between the overexpressed genes in both populations with no-predictive significance between ER or NR, one of them, namely* INSL4 -* which presents an overexpression in an elevated number of patients examined* -* was investigated in detail. Although INSL4 is produced by invasive breast cancer cells and is co-expressed with HER-2 in a cluster of cells in the invasive front of neoplasia 11, 12, little is known about involvement of INSL4 in NSCLC.

The relaxin/insulin-like (RLN/INSL) gene family comprises a group of signalling molecules that have physiological roles mostly related to reproduction and neuroendocrine regulation. INSL family peptides are secreted molecules acting in an autocrine or paracrine fashion, to engage RelaXin Family Peptide (RXFP) receptors, which, in turn, trigger multiple signalling pathways. Receptor engagement by the peptide and the resulting signalling pathway give rise to a variety of cellular responses, mostly dictated by the nature of the tissue.

We explored INSL4 function in NSCLC adenocarcinoma cells by using a Gain-of-Function approach, which is an excellent/benchmark tool for assessing the functional importance of cancer-related genes. In an *in vitro* setting, NSCLC adenocarcinoma H1299 cell line was used as a mean of identifying signalling pathways, which - upon activation by INSL4 overexpression - might affect cell growth and drug responsiveness. *In vivo,* the effect of INSL4 overexpression was studied in a xenograft mouse model. In addition to H1299 we used another lung cancer cell line A549, to explore whether INSL4 overexpression improves the growth. Comparing the two different cell lines we found a similar value in cell growth, while Colony-formation assay and soft-agar assay did not show differences respect to the control. Analysis in mRNA expression of INSL4 instead, showed a huge discrepancy between *INSL4* gene expression and cell growth. Later, we analysed the divergences in eight different LC cell lines defining that *INSL4-mRNA* is highly regulated at post-transcriptionally/post-translationally level. Thus, we analysed expressed levels of *INSL4* in patients with NSCLCs adenocarcinoma. A significant discrepancy was found to occur between levels of *INSL4-mRNA* expression and protein content. Moreover, *in silico* analysis for assessing patients whose NSCLCs adenocarcinoma spontaneously expressed high levels of *INSL4*, a significant inverse correlation was found to occur between levels of *INSL4* expression with poor Overall Survival (OS). By combining the results obtained from the *in vitro* and *in vivo* models and patients our results provide evidence for a potentially important role for INSL4 in the biology of adenocarcinoma NSCLC.

## Materials and Methods

### Plasmid constructions and cloning

INSL4-encoding sequences were amplified by PCR and cloned in pSCB using the Strataclone Blunt PCR Cloning Kit (Agilent, Santa Clara, CA, USA), according to manufacturer's instruction using specific primers for *INSL4*. Forward primer: 5'-CCCTCGAGCCCATGGCCAGCCTGTTCCGGTCC-3'; reverse primers: 5'-CCCTCGAGCCCTGTACATAATTTAACTGAAGTTCCATC-3' and 5'-CCCTCGAGCCCTATGTACATAATTTAACTGAAGTTCCA-3'. Construct was then subcloned into the pcDNA3.1 expression vector carrying a Myc-Tag sequence to obtain a Myc-tagged INSL4 fusion protein.

### Cell culture and treatment

Human Non-Small Cell Lung Carcinoma H1299, A549, H1650, H1975, H460, HCC827, CALU-1 and CALU-3 cell lines were maintained in RPMI 1640 medium (Euroclone, Milan, Italy) containing 10% fetal bovine serum (FBS; Euroclone). H1299, A549 and H460 cells were transfected with Myc-tagged INSL4 expressing or control vector, H1299 using Fugene6^®^ (Promega Corporation, Madison, WI, USA), A549 using Lipofectamine LTX (Invitrogen, Carlsbad, CA, USA), according to manufacturer's instructions. Stable H1299 and A549 cell lines were selected *via* the addiction of geneticin (0.4 mg/ml) (Sigma-Aldrich, St. Louis, MO, USA) to culture medium. Cell growth rates were determined as previously described 13. Briefly, H1299- and A549-INSL4 and relative control cells were seeded at a concentration of 1.5 × 10^5^ cells/well. Cell numbers were counted at 1, 2, 3 and 4 days after seeding using a Burker haemocytometer by trypan blue (Euroclone) exclusion. All the experiments were performed in triplicate at least three times.

### RNA extraction and Quantitative Real-Time PCR (qPCR)

The study protocol was approved by the local Ethics Committee and was conducted in accordance with the ethical principles of the latest version of the Declaration of Helsinki. Written informed consent was obtained from all patients before enrolment. Tumour tissue specimens were collected from patients diagnosed with AC-NSCLC. RNA extraction and qPCR were performed as previously described 14. Total RNA was extracted from tumour tissues using TRIzol^®^ (Invitrogen) and from cell lines using NucleoZOL^®^ (Macherey Nagel, Duren, Nordrhein-Westfalen, Germany), according to the manufacturer's instructions. cDNA was retrotranscribed from 1 µg of RNA using the iScript kit (BioRad, Hercules, CA, USA). qPCR was performed with SYBR^®^ Green qPCR Master Mix (Invitrogen) using INSL4-specific primers: forward 5'-GCTGCTGAGCCAACTCCTTAG-3', reverse 5'-GGGTGGTGGTGAATGTCTTCTC-3'. The relative amount of mRNA was normalized to human hypoxanthine phosphorybosiltransferase (HPRT). Forward 5'-CGAGATGTGATGAAGGAGATGGG-3', reverse 5'-GATGTAATCCAGCAGGTCAGCAA-3'.

### Western blotting analysis and antibodies

Western blotting analysis was performed with protein extracts lysed in Laemmli buffer (Tris-HCl 200 mM at pH 6.8, SDS 8%, bromophenol blue 0.4%, glycerol 40% and β-mercaptoethanol 5%) under denaturing condition and boiled for 5 minutes. Proteins were resolved on SDS-polyacrylamide gel and transferred by electroblotting onto nitrocellulose membranes. Blots were blocked in non-fat dry fat-free milk 5% in PBS (Sigma-Aldrich) for 1 hour and then incubated overnight with the primary antibodies at 4°C. Primary antibodies were anti Myc-tag (9E10), AKT, phosphorylated AKT (Ser473), p44/42 MAPK, phosphorylated p44/42 MAPK (Thr202/Tyr204) were all purchased from Cell Signaling (Danvers, MA, USA). Anti-tubulin antibody and phalloidin-FITC conjugate were from Sigma-Aldrich. Detection was achieved using Goat anti-Rabbit and anti-Mouse IgG (H+L) HRP conjugate secondary antibodies from BioRad (Hercules, CA, USA) and visualized with ECL (GE Healthcare, Lifescience, Little Chalfont, UK).

Experiments were performed at least three times and performed each time in triplicate.

### Microscopy Analysis

Immunofluorescence analysis was performed in H1299 cells fixed with 4% paraformaldehyde (Sigma-Aldrich), permeabilized with Triton X100 0.2% (Sigma-Aldrich) and blocked with 3% BSA (all from Sigma-Aldrich). Cells were stained with anti-Myc-Tag (9E10, Sigma-Aldrich) - for INSL4 analysis - and anti-calreticulin (StressGen Biotechnologies Corp., BC, Canada) antibodies. Goat anti-Mouse IgG (H+L) Alexa Fluor^®^ 555 conjugate and Goat anti-Rabbit IgG (H+L) Alexa Fluor^®^ 488 conjugate secondary antibodies were used respectively (Thermo Fisher Scientific, Waltham, MA, USA). DNA was stained with DAPI (Sigma-Aldrich). Cells alternatively stained with a fluorescent phalloidin-FITC conjugate (Sigma-Aldrich) solution in PBS at room temperature for 40 minutes. Phalloidin-FITC conjugate were used as a cytoskeleton marker.

Fluorescence analysis was performed using Zeiss Axioplan (Zeiss, Oberkochen, Germany) fluorescence microscope controlled by Spot-2 cooled camera (Diagnostic Instruments, Sterling Heights, USA). Experiments were performed at least three times and performed each time in triplicate. Images were exported in TIFF, and contrast and brightness were adjusted in Corel Paint Shop Pro X9 and final figures were generated with Adobe Illustrator 6.

### FACS analyses

Cell cycle analysis was carried out by flow cytometry using FACStar Plus flow cytometer (Becton Dickinson, Franklin Lakes, NJ, USA) with propidium iodide (PI) (Sigma-Aldrich) staining. Cells were collected and adjusted to a concentration of 1 × 10^6^ cells/ml and fixed in 70% ethanol at 4 ºC for 30 minutes. Cells were washed twice with cold PBS, once with BSA 1% in PBS and then incubated for 30 minutes with RNase (250 μg/ml) (Sigma-Aldrich) and PI (50 μg/ml).

Experiments were performed at least three times and performed each time in triplicate.

### Colony formation assay

For Colony formation assay cells were seeded at 5 × 10^2^ in 6-well dishes and the following day, complete medium was replaced with RPMI 1640 + 5% FBS and cells were grown for 2 weeks. At the endpoint, plates were fixed with 4% formaldehyde (Sigma-Aldrich) and stained with 2% crystal violet (Sigma-Aldrich). Finally, the plates were used for image acquisition with a digital camera. Experiments were performed at least three times and performed each time in triplicate.

### Soft agar colony formation assay

Anchorage-independent growth of tumour cells was estimated by a soft agar colony formation assay. In brief, 1,2% base agar in RPMI-1640 complete medium was polymerized in 6 wells Petri dishes to make a growth substrate. Cells (5 × 10^3^/well) were quickly resuspended in agarose (1%) in complete medium and the mix was carefully stratified onto the base agar. Foci were allowed to develop for a 10-14-day incubation. Twenty fields were selected for each cell condition to count Colony Forming Units (CFU; 1 CFU ≥ 50 cells) under an inverted microscope.

Experiments were performed at least three times and performed each time in triplicate.

### Xenografts mouse model

NOD/SCID mice were obtained from Charles River (Wilmington, MA, USA). Mice were maintained under specific pathogen-free conditions in ventilated (high-efficiency particle-arresting filtered air) sterile microisolator cages (Techniplast, Milan, Italy) at constant temperature (24-26 °C), constant humidity (30-50%), and a 12-hours light/12-hours dark cycle. Sterilized food and tap water were given *ad libitum*. Exponentially growing H1299-INSL4 and H1299-control cells were harvested and washed twice with PBS before resuspending in PBS. Xenograft tumour implants were established in NOD/SCID male mice (6-8 weeks old) by subcutaneous injection (4 × 10^6^ cells /200 µl PBS) in the right flank. Tumour size was measured with a caliper each 5 days and volumes were estimated using the formula: length (mm) × width (mm) × height (mm)/2. Animals were sacrificed 4 weeks after tumour cell implantation and tumour was removed, weighed, and split into 2 parts, one to be fixed in 10% buffered formalin for histological analysis, and the other to be snap-frozen in liquid nitrogen and stored at -80 °C for molecular analysis.

### Immunohistochemistry

Histological analysis was performed as previously described 15. Briefly, paraffin-embedded tumour tissues were sectioned (4 μm) and stained by haematoxylin and eosin (H&E), to observe tissue structure and calculate mitotic index values by enumerating proliferating cells. For the Ki67 index, the tissue section slides were blocked and incubated with specific anti-Ki67 antibody (Clone Mib1, DAKO; Agilent, Santa Clara, CA, US).

### Tissue Microarray

Tissue microarrays were prepared as previously described 16. Overall, 2-mm cores were obtained from the most representative areas of the tumours and then re-embedded in microarray blocks. Each case was sampled 3 times. Prior to tissue microarray construction, an H&E slide of each block was analysed to exclude non-representative inappropriate regions (e.g. necrosis and haemorrhage).

Immunohistochemistry analysis was performed on 4-μm-thick sections from each formalin-fixed and paraffin-embedded tissue microarray block. For antigen retrieval, sections were heat-treated for 30 min. Tissue sections were incubated with a specific polyclonal anti-INSL4 primary antibody (Abnova, Taipei City, Taiwan) for 15 min. The slides were processed with the Bond-Max™ (Vision BioSystems, Norwel, MA, USA) automated slide preparation system, and the Bond™ Polymer Refine Detection (Vision BioSystems) was used as an antibody detection system. Appropriate negative (NL) and positive (placenta) control tissues slides were processed concurrently.

### *In silico* structural and functional analyses of INSL4

To define INSL4 protein domains we examined the UniPRO and ExPASy-PROSITE protein databases (http://www.uniprot.org/, http://prosite.expasy.org/).

In order to analyse the presence of putative cleavage sites and functional linear motifs in the primary sequence of the protein we consulted the SignalP 5-0 (http://www.cbs.dtu.dk/services/SignalP/) and the Eukaryotic Linear Motif databases (http://www.cbs.dtu.dk/services/SignalP/, http://elm.eu.org).

### *INSL4* expression and prognostic effect of INSL4 in NSCLC patients' publicly available datasets

Analysis of pattern of *INSL4* expression in normal and tumour tissues was performed using the Affymetrix U133Plus2.0 platform (medical-genome.kribb.re.kr/GENT). *INSL4* expression was assessed using the GENT2 (Gene Expression database of Normal and Tumour tissues 2) database (http://gent2.appex.kr/gent2/).

Screening to evaluate *INSL4* overexpression in LC patients was performed using COSMIC (Catalogue Of Somatic Mutations In Cancer) (http://cancer.sanger.ac.uk/cosmic). The Kaplan Meier Plotter (http://kmplot.com) 17 was used to assess the association of *INSL4* mRNA expression with clinical endpoints.

### Statistical analyses

Data are presented as means ± SEM, unless otherwise indicated in figure legends. Sample number (*n*) indicates the number of independent biological samples in each experiment. Statistical analysis of experimental data was performed using Student's *t* test or paired *t* test. Data analysis was not blinded. Significance levels are: *P < 0.05; **P < 0.01; ***P < 0.001. Analyses were performed using the Excel software.

### Ethics approval and consent to participate

The study was approved by the local Ethics Committee *Comitato Etico delle Aziende Sanitarie della Regione Umbria (CEAS)* and was conducted in accordance with ethical principles of the latest version of the Declaration of Helsinki. Written informed consent for gene expression analyses was obtained from each patient entering the study. All experiments involving animals were authorized by Ethics Committee *of Ministero della Salute Direzione Generale della Sanità Animale e dei Farmaci Animali* and were done according to the guidelines of the *University of Perugia Ethical Committee* and the *European Communities Council Directive* 2010/63/EU.

## Results

### *INSL4* structure analysis and localization in NSCLC

**Figure 1:**
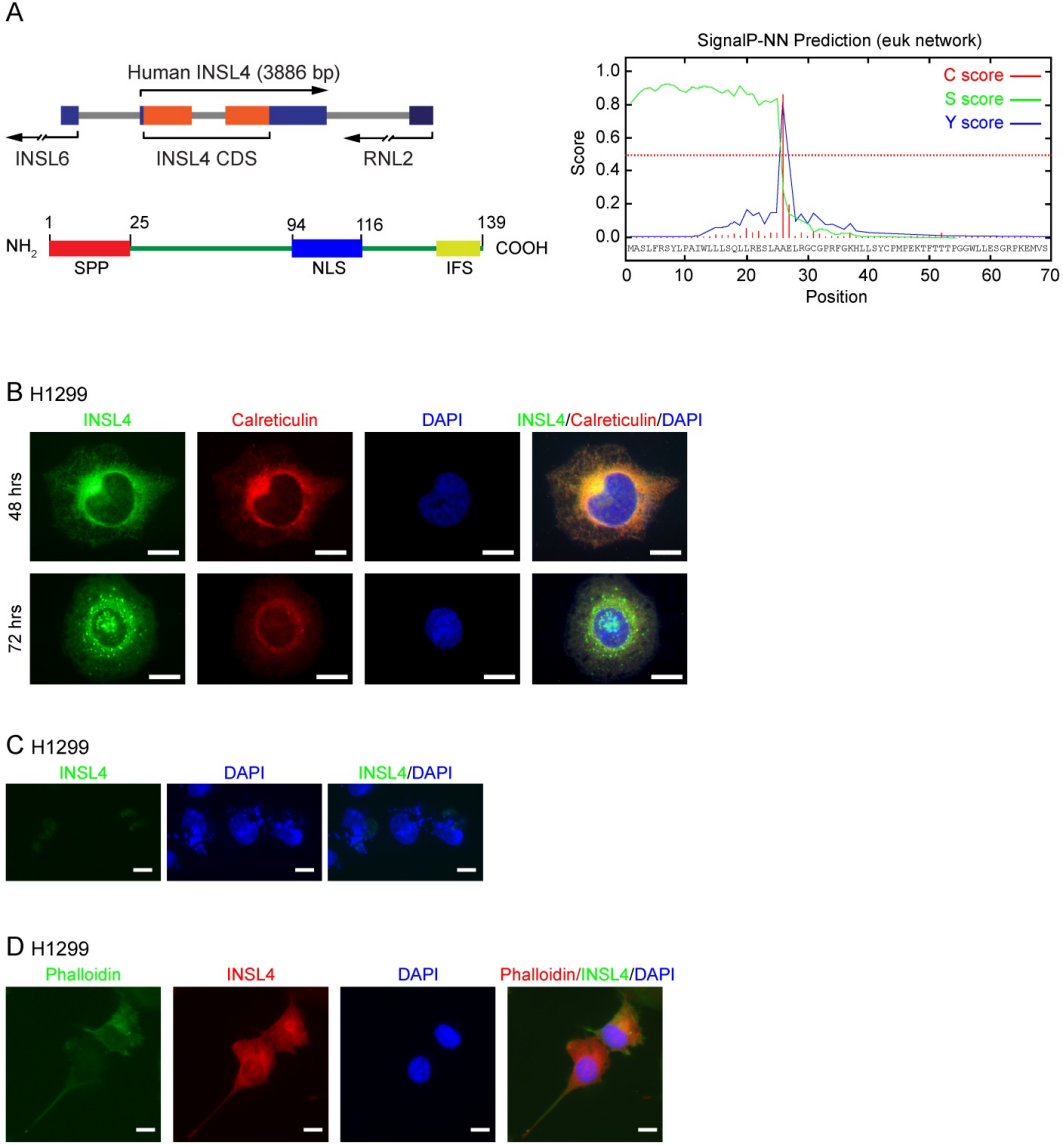
** INSL4 structure and localization in NSCLC H1299. A,** Schematic diagram of INSL4 gene and protein (left) and bioinformatics prediction for SignalP-5.0 (right). **B,** Representative images of the immunofluorescence analysis of INSL4 transfected H1299 cells 48 or 72 hours after transfection. Cells were fixed and stained with antibodies to the fusion protein INSL4-Myc-Tag (INSL4), and calreticulin and revealed using the appropriate secondary antibodies. Nuclei were DAPI stained. **C,** Representative images of the immunofluorescence analysis of not permeabilized INSL4 transfected H1299 cells at 72 hours of transfection. Cells were stained using antibody to INSL4-Myc-Tag (anti myc-Tag) as previously. Nuclei were DAPI stained. Samples were analyzed under fluorescent microscope. **D,** Representative images of the immunofluorescence analysis on INSL4 transfected H1299 cells labeled using phalloidin-FITC conjugate (Phalloidin) as cytoskeleton marker and anti Myc-Tag for INSL4 detection. Nuclei were DAPI stained. Samples were analyzed under fluorescent microscope. Representative images of three independent experiments are shown. Scale bars 10 µm.

*INSL4 gene* was located on human chromosome 9p24, with an open reading frame (ORF) of 417 bp coding for a 139 amino-acid protein 18, 19. Using UniPRO and ExPASy-PROSITE protein databases, we attempted to define INSL4 protein domains. We found a Signal-Peptide Peptidase (SPP) cleavage-site located at the N-terminus and an Insulin Family Signature (IFS) at the C-terminus (Figure 1A, left). Cleavage by SPP was found to occur between amino acids at positions 25 and 26 (Figure 1A, right). The* in silico* study to determine INSL4 putative localization revealed that INSL4 is a secretory protein mostly restricted to the extracellular compartment. This suggested that INSL4 is cleaved and modified in the Golgi apparatus, where it acquires secretory protein features. Indeed, a short putative Nuclear Localization Site (NLS) has been described between Aa 94-116.

We studied INSL4 localization in H1299 cells by cloning *INSL4* in an expression vector, so to generate a fusion protein with the Myc epitope sited in the protein C-terminus. On immunofluorescence analysis, INSL4 was not detectable across the whole cell body, but rather restricted to the Golgi and Endoplasmic Reticulum (ER) compartments, as revealed by immunolocalization with specific antibody and calreticulin respectively, the latter being used as a marker of subcellular district (Figure 1B). To check for any possible changes in intra and extracellular INSL4 localization, as suggested by the *in silico* analysis of INSL4, we examined H1299 cells for INSL4 expression at 48 and 72 hours of transfection. The longer term at 72 hours provided evidence by immunofluorescence images for the presence of INSL4 in vesicles and granules of secretion (Figure 1B). We thus analysed INSL4 localization in not permeabilized cells at 72 hours of transfection. INSL4-transfected cells were characterized by the obvious presence of INSL4 in the peri- and extra-cellular regions (Figure 1C). Moreover, staining the actin cytoskeleton with phalloidin-FITC conjugate at 72 hours of transfection revealed INSL4 as being localized in actin-rich membrane protrusions (Figure 1D). These results substantiated the data, predicted by the *in silico* observations, being likewise in accordance with previous data on INSL4 secretion and its distribution in autocrine compartments 12, 20, 21.

### INSL4 promotes proliferation and invasiveness by NSCLC

**Figure 2:**
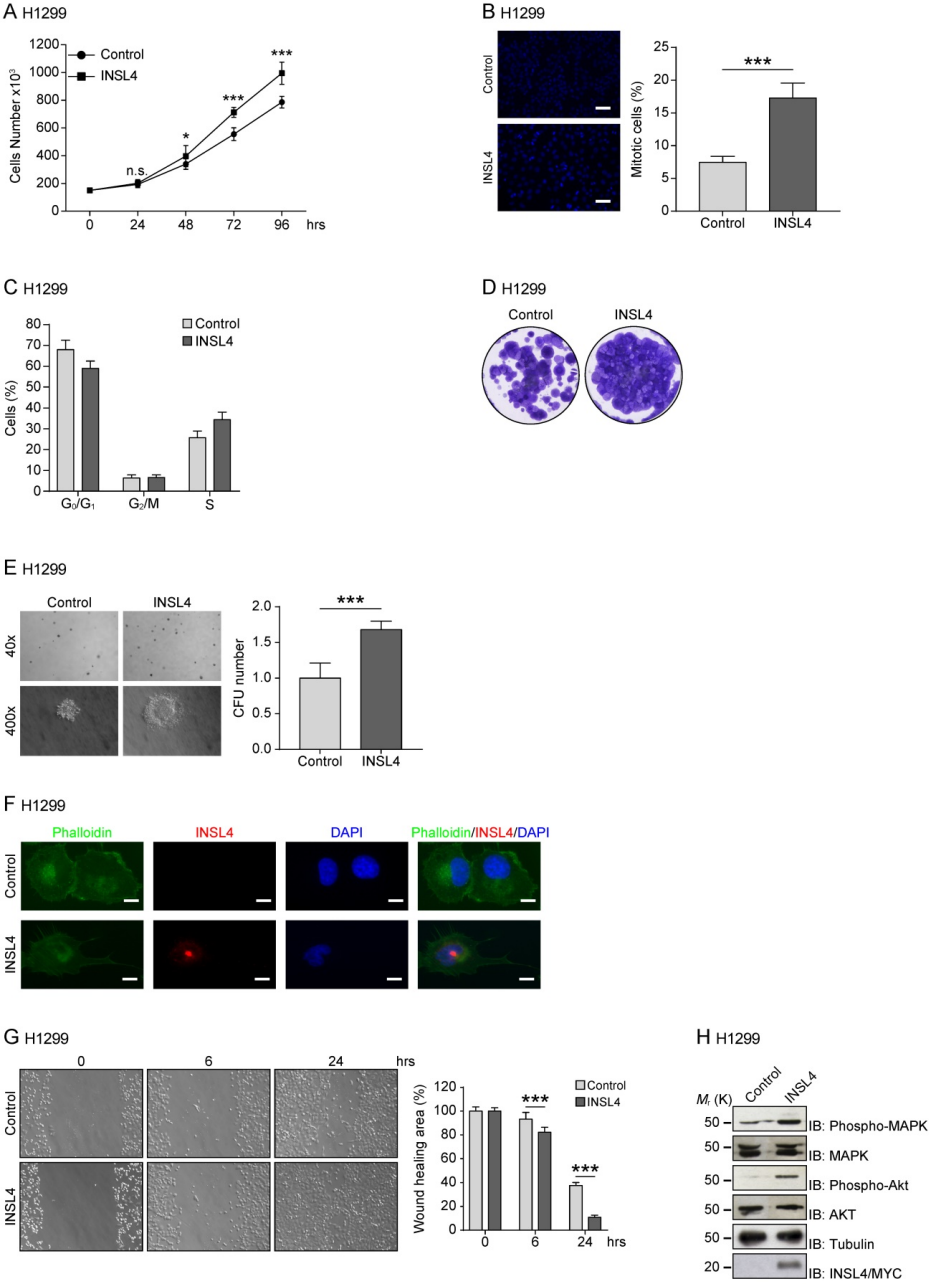
** INSL4 role in NSCLC proliferation and invasiveness in H1299 cells.** Representative images of each analysis are shown. **A,** Cell growth assay was performed in H1299 stably expressing a control vector (Control) and H1299-INSL4 (INSL4) cells. **B,** Mitotic index in H1299 (Control) and H1299-INSL4 cells (INSL4). Two hundred cells were scored for mitoses by immunofluorescence analysis. Results were shown as percentage of mitotic cells. Results are presented as means ± SEM. Nuclei were DAPI stained. Scale bars 10 µm. **C,** Cell cycle profiles of H1299 (Control) and H1299-INSL4 cells (INSL4) determined by FACS analysis. Graph illustrates the cell percentage in the different cell cycle phases. Results are presented as means ± SEM. **D,** Colony formation assay in H1299 (Control) and H1299-INSL4 cells. Cells were stained with crystal violet. **E,** Soft agar assay in H1299-control and H1299-INSL4 cells. The graph illustrates the score of twenty randomly selected fields. **F,** Immunofluorescence analysis in H1299 (Control) and H1299-INSL4 cells was performed using phalloidin-FITC conjugate (Phalloidin) as cytoskeleton marker and anti-Myc-Tag for protein INSL4-Myc-Tag detection (INSL4), and revealed using the appropriate secondary antibodies. Nuclei were DAPI stained. Scale bars 10 μm. **G,** Wound healing assay in H1299 (Control) and H1299-INSL4 cells (INSL4). The graph illustrates the percentage of residual wound healing area at 0, 6 and 24 hours from injury. **H,** Analysis of activation signaling pathways for proliferation was performed by western blotting on H1299 (Control) and H1299-INSL4 cells using the indicated antibodies. Results are presented as means ± SEM. *, P<0.05; **, P<0.01; ***, P <0.001. All the images are representative of three independent experiments.

To study the functional significance of INSL4 in NSCLC, we generated INSL4 stably overexpressing H1299 cells (hereafter referred to as H1299-INSL4). As a control, H1299 cells were transfected with the empty vector (Control) (Supplementary Figure S1A). Growth rates were simultaneously observed. At 96 hours of culturing, an increase approximately 27% was found in the proliferative index of H1299-INSL4 cells relative to controls (*P* < 0.001) (Figure 2A). Next, we analysed mitotic indexes by enumerating mitoses in H1299-INSL4 and H1299-control cells. In INSL4-overexpressing cells, the mitotic index increased by 2.5-fold value relative to controls (Figure 2B). Consistently, cell cycle progression observed by FACS analysis showed that INSL4 promotes a reduction of cells in G_0_/G_1_ phase and an increase of those in S phase (Figure 2C).

To further substantiate the effect of INSL4 on cell proliferation, we investigated the behaviour of INSL4 overexpressing cells, in a colony-forming assay. Clonogenic assays were performed using H1299 cells as control and H1299-INSL4. Cells were cultured for 2 weeks under low-serum conditions before assessing their ability to generate colonies. Significant differences in growth rates of H1299-INSL4 compared to control cells were observed. INSL4 overexpression conferred to transfected cells an increased ability to generate colonies with enhanced cellular density relative to controls (Figure 2D). To better document the impact of INSL4 on cell proliferation, we performed a soft agar assay using H1299-INSL4 and H1299-control cells. INSL4 overexpression significantly increased the ability of H1299 cells to form colonies in soft agar (Figure 2E). Indeed, the analysis of the soft-agar plates showed not only a marked increase in the total number of colonies generated by INSL4, but those overexpressing INSL4 were characterized by a more consistent and fringed pattern relative to controls (Figure 2E).

Another important finding from fluorescent-microscope analysis of H1299-INSL4 cells stained with phalloidin was the observation that INSL4 overexpression modifies cell morphology. H1299-INSL4 cells displayed large and numerous protrusions not found in control cells. Moreover, the protrusions of cellular edges appeared thin and long (Figure 2F). This data strongly suggested that INSL4 increases invasiveness in NSCLC. We asked whether those changes were accompanied by changes in cell motility. Scratch assays were performed *in vitro* to examine the effects of cell-matrix and cell-cell interactions on cell migration. Comparative analysis of H1299-INSL4 and control cells showed a clear difference in motility patterns between the two populations, indicating INSL4 may indeed increase NSCLC motility (Figure 2G).

Because the release of INSL4 from cells could have autocrine and paracrine effects, we studied the pathways exploited by the insulin-like factor to activate cell growth. Although a class of receptors have been identified for INSL peptides, namely, the RXFP receptor family, the specific nature of the receptor for INSL4 has yet to be defined. We investigated the ability of INSL4 to trigger the MAPK pathway, one of the most important signalling pathway involved in cell proliferation 22, 23. Western blotting analysis of H1299-INSL4 and control cells revealed an increased MAPK phosphorylation in the INSL4 overexpressing population. Similar phenotype was observed analysing AKT phosphorylation (Figure 2H). The AKT signalling pathway plays a central role in many cellular processes, and it contributes to cancer progression 24.

### INSL4 overexpression leads to increased tumour growth *in vivo*

**Figure 3:**
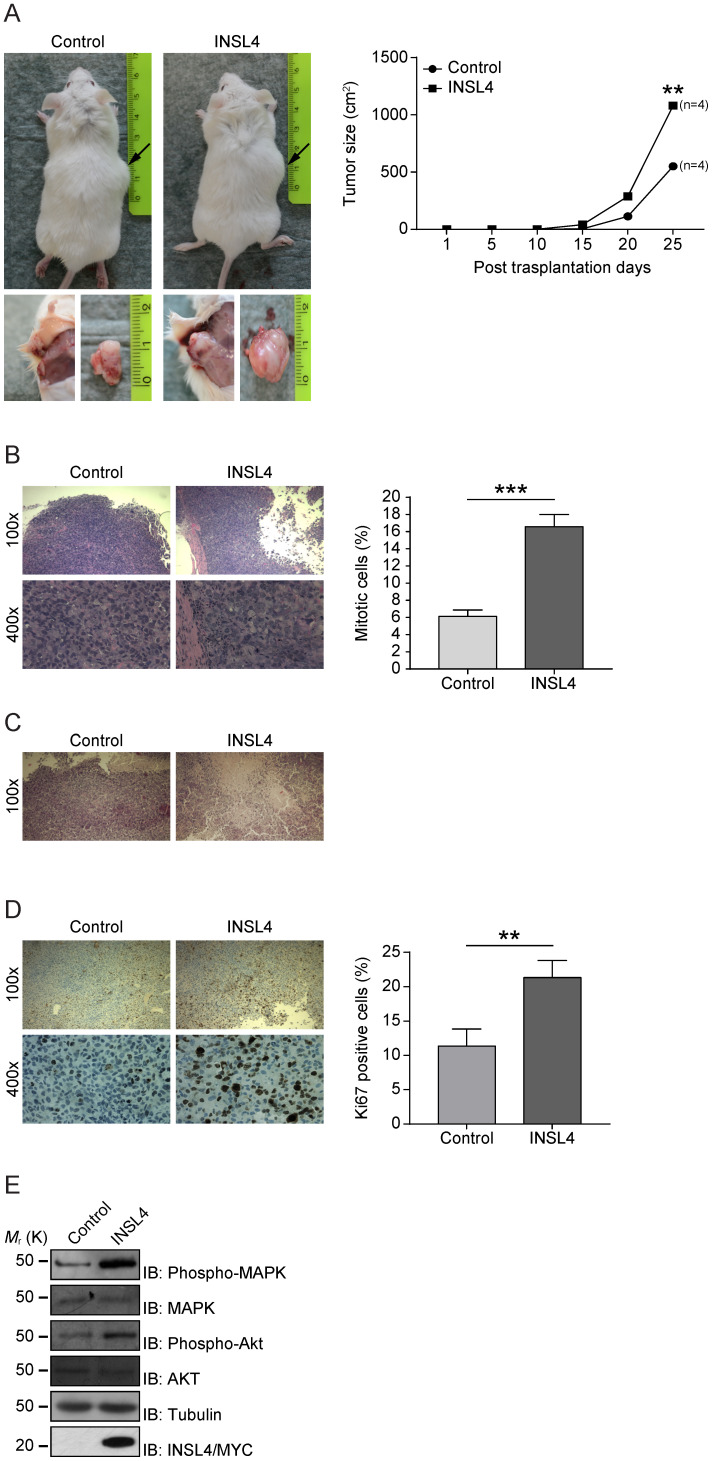
** INSL4 influence tumour growth. A,** H1299 cells engraftment in mice. NOD/SCID mice (n=4 per group) were subcutaneously injected using H1299 (Control) or H1299-INSL4 (INSL4) cells as described. Tumour growth progression is displayed in graph. Volume of each mass (n=4 per condition) is recorded every five days after cellular injection and reported in graph as means ± SEM. Representative pictures showed the external view of tumour-bearing NOD/SCID mice and the *in situ* and post-explantation tumour masses. **B,** H&E staining of xenograft tumour. Thirty randomly selected slides were scored for mitoses. The shows the mitotic index. The results are presented as means ± SEM of percentage of mitotic cells. **C,** H&E staining of xenograft tumors shows extensive necrotic areas in INSL4 masses relative to Control. Scale bars 10 µm. **D,** Ki67 staining in xenograft tumors. The graph displays the percentage of Ki67 positive cells. Thirty randomly selected slides were scored. Results are presented as means ± SEM. E, Pathways activation in xenograft induction was performed by Western Blotting analyses in lysates from tumour masses using the indicate antibodies. *, P<0.05; **, P<0.01; ***, P <0.001. All the images are representative of three independent experiments.

Because INSL4 has a functional *in vitro* effect in NSCLC proliferation and invasion, we investigated any contributions of INSL4 to tumour growth *in vivo*. Control H1299 and H1299-INSL4 were injected into NOD/SCID mice to study the proliferative potential of the cell lines. Tumours size was measured at 5 day intervals after injection and all mice were sacrificed on day 25 after engraftment. Mouse grafting with H1299-INSL4 cells resulted in a 2-fold increase in growth rates relative to control xenografts, as demonstrated by tumour growth kinetics. Tumour xenografts of both cell types showed similar growth patterns until day 10, when a difference became instead appreciable. Endpoint analyses of tumour weights showed a 50% increase in tumour masses of H1299-INSL4 recipient, relative to control mice (Figure 3A). Tumour growth was indeed obvious on gross inspection of H1299-INSL4 tumour-bearing hosts, with histopathology showing extensive vascularization as well as infiltration of muscular tissues and bones in rib cages. Histopathology further revealed poorly differentiated H1299-INSL4 cells, whose morphology was marked by the widespread presence of mitoses. On enumerating mitotic cells in both types of tumour, a 2.7-fold increase was found in INSL4-overexpressing masses (Figure 3B). Moreover, the H1299-INSL4 tumour masses present necrotic areas, which were instead absent in control specimens (Figure 3C). A 2-fold higher labelling index of Ki67 - a hallmark of cell proliferation - was found in the latter cells (Figure 3D). Likewise, in the *in vitro* setting, we detected MAPK and AKT activation in extracts from INSL4-overexpressing tumour masses (Figure 3E).

**Figure 4:**
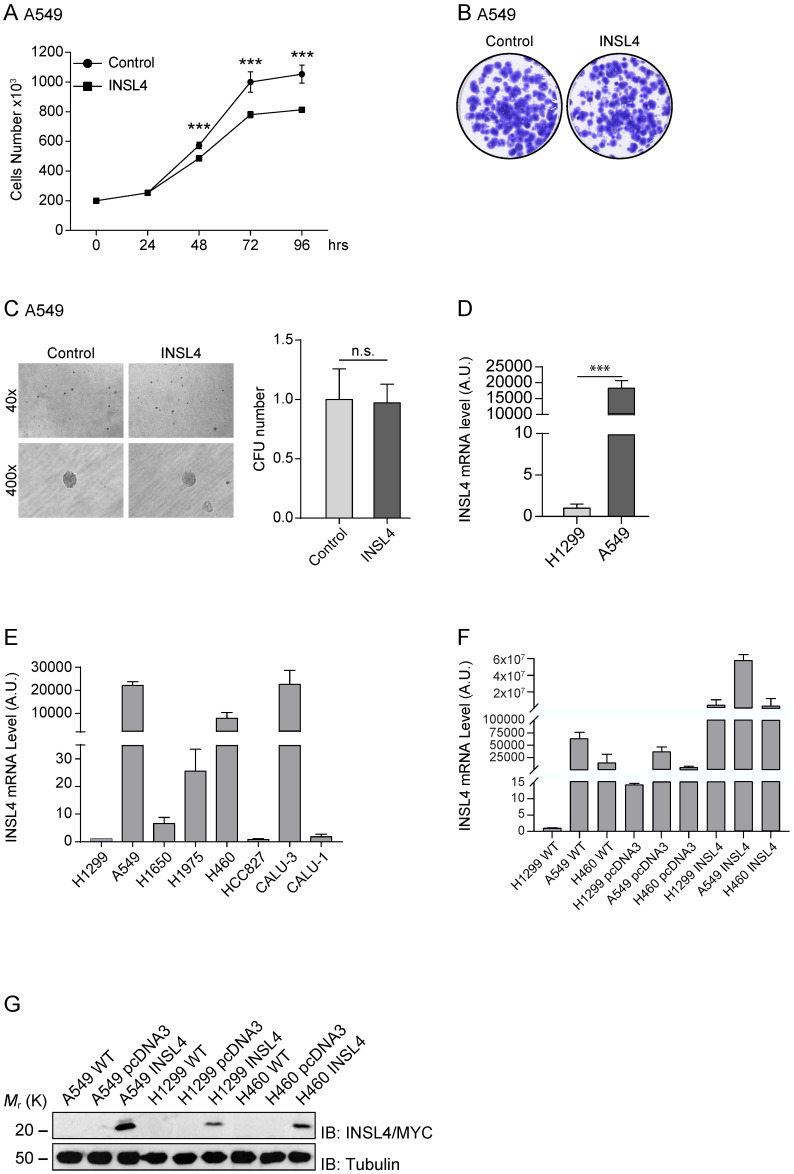
** INSL4 role in NSCLC proliferation and invasiveness in A549 cells and INSL4 mRNA and INSL4-Myc-Tagged protein expression. A,** Cell growth assay was performed in A549 stably expressing a control vector (Control) or INSL4 (INSL4). **B,** Colony formation assay in A549-control and A549-INSL4 cells. Cells were stained with crystal violet. **C,** Soft agar assay in A549 (Control) and A549-INSL4 cells (INSL4). The graph illustrates the score of twenty randomly selected fields. Results are presented as means ± SEM. *, P<0.05; **, P<0.01; ***, P <0.001. All the images are representative of three independent experiments. **D,** INSL4 mRNA levels were studied using qPCR analysis in the reported H1299 and A549 cells assuming the amount of INSL4 mRNA of H1299 as Arbitrary Unit (A.U.) 1 (left). INSL4 protein levels on the same NSCLC cell lines were studied by immunoblot analysis using anti-INSL4 antibody. Anti-Tubulin antibody is loading used as control. In graphical representation relative H1299 INSL4 protein level was assumed as Arbitrary Units (A.U.) 1 (right). **E,** INSL4 mRNA levels were studied using qPCR analysis in the reported tumour cell lines assuming the amount of INSL4 mRNA of H1299 as Arbitrary Unit (A.U.) in the graph. **F,** Levels of endogenous and transfected INSL4 mRNA in H1299, A549, H460 NSCLC cell lines transfected with fusion protein INSL4-Myc-Tag and pcDNA3.1 (control), the absolute mRNA levels were compared to mRNA levels in H1299 WT. G, INSL4 protein levels on the same NSCLC cell lines showed in F were studied by immunoblot analysis using anti-Myc antibody. All the images are representative of three independent experiments.

To verify the important role of INSL4 in LC, we further analysed, the A549 cell line. We stably transfected A549 cells with the fusion protein INSL4-Myc-Tag, (hereafter referred to as A549-INSL4) and the empty vector as control (Control). The INSL4 expression was controlled by Western Blotting analysis (Supplementary Figure S1B) and growth rate was assessed. Unexpectedly, at 72 hour of culturing, a decrease in the proliferative index around of 50% was found in A549-INSL4 cells relative to controls (Figure 4A).

After this unpredicted result, we investigated other proliferative indicators in A549-INSL4 cells such as colony-forming assay and soft agar. Clonogenic assay was performed in A549-INSL4 and control cells. As expected, no significant differences in growth rates of A549-INSL4 compared to controls were observed (Figure 4B). Then, we performed soft agar assay in A549-INSL4 and control cells. Also in this case, INSL4 overexpression did not increase the number and the size of colonies in soft agar (Figure 4C).

After these results, we asked why this discrepancy in growth rate between H1299 and A549 was present. Indeed, comparing the basal mRNA expressed in the H1299 and A549, we found that *INSL4-mRNA* is highly expressed in A549, almost 22×10^3^ times respect to H1299 (Figure 4D left). At this point, we analysed eight different tumour cell lines of NSCLC. We explored the mRNA content of *INSL4* in: H1299, A549, H1650, H1975, H460, HCC827, CALU-3 and CALU-1 assuming the amount of *INSL4-mRNA* of H1299 as Arbitrary Unit 1. The data obtained show that in three cell lines on eight, in particular A549, H460 and CALU-3 we found a huge increase in *INSL4-mRNA* with values of 22262, 7882 and 23198 respectively compared to H1299 (Figure 4E). The discrepancy observed between the mRNA and proliferation, mostly observed in in A549 cell lines, has prompted us to determine whether the *INSL4 mRNA* has a control in translation. To do this, we transfected the NSCLC cell lines A549, H1299 and H460 with INSL4 fused with Myc epitope in C-terminus, then we analysed the level of mRNA and the protein content produced. In particular, we found, as expected, high level of INSL4-mRNA in all the three cell lines analysed by qPCR (Figure 4F). Western Blot analysis was performed by using an anti-Myc to detect the INSL4 protein. Notably, although the high differences in mRNA amount, the protein content in all the LC cell lines showed little differences among them (Figure 4G), a huge discrepancy is present between the amount of mRNA and protein in the cell lines.

### INSL4 analysis in NSCLC patients

**Figure 5:**
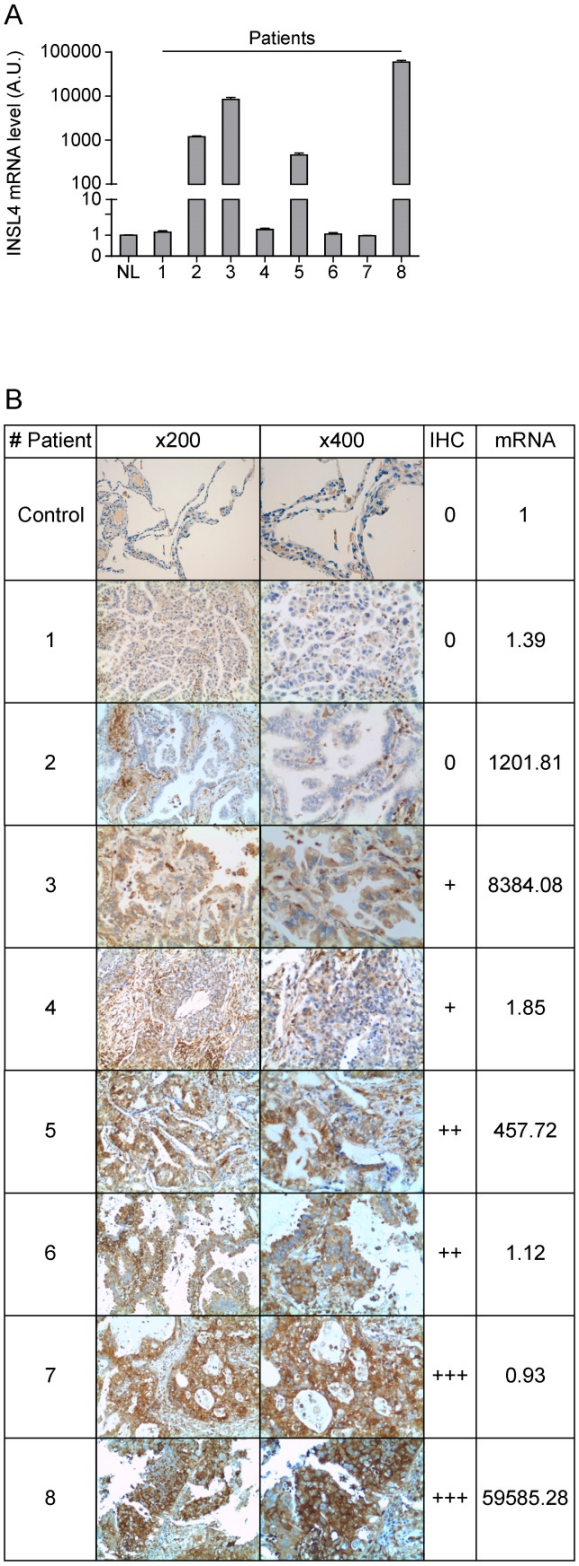
** INSL4 mRNA and protein expression in lung cancer tissues. A, I**NSL4 mRNA levels were studied using qPCR analysis in the specimens of patients (indicated 1 to 8) with NSCLC and in normal lung (NL) assuming the amount of INSL4 mRNA as an Arbitrary Unit (A.U.) 1. **B,** Immunohistochemistry for INSL4 protein levels in the specimens examined for qPCR analysis. Samples were categorized with different grade of positivity for immunohistochemistry. Immunohistochemistry range of positivity is from 0 to +++, NL is used for control. INSL4 mRNA values were reported in the last right column and refer to the images of relative histological samples. Magnification x200 and x400. INSL4 mRNA values were reported in the last right column and refer to the images of relative histological samples. Magnification x200 and x400.

Because the divergence between transcription and translation of INSL4 detected in LC cell lines examined, we compared mRNA expression and protein content in a cohort of patients with NSCLC. In accordance with previously reported gene expression profiles, we examined a cohort of patients with AC-NSCLC 10. *INSL4-mRNA* expression of eight individual patients was determined by qPCR analysis. *INSL4-mRNA* levels were significantly up-regulated in four NSCLC patients out of the eight being examined, relative to normal lung tissues from healthy subjects (Figure 5A).

Importantly, the histological analysis of the specimens shows similar results to those observed in LC cell lines. In patients with NCLSC we observed that a high expression of *INSL4-mRNA* does not always correlate with a high amount of protein, while in some patients with low *INSL4-mRNA* a great INSL4 protein content is present (Figure 5B and Supplementary Figure S2). These data indicate that there is no linear correlation between expression of the *INSL4* gene and protein, but an individual epigenetic control in each single patient should be strongly considered.

### *INSL4* overexpression predicts poor survival in NSCLC patients

**Figure 6:**
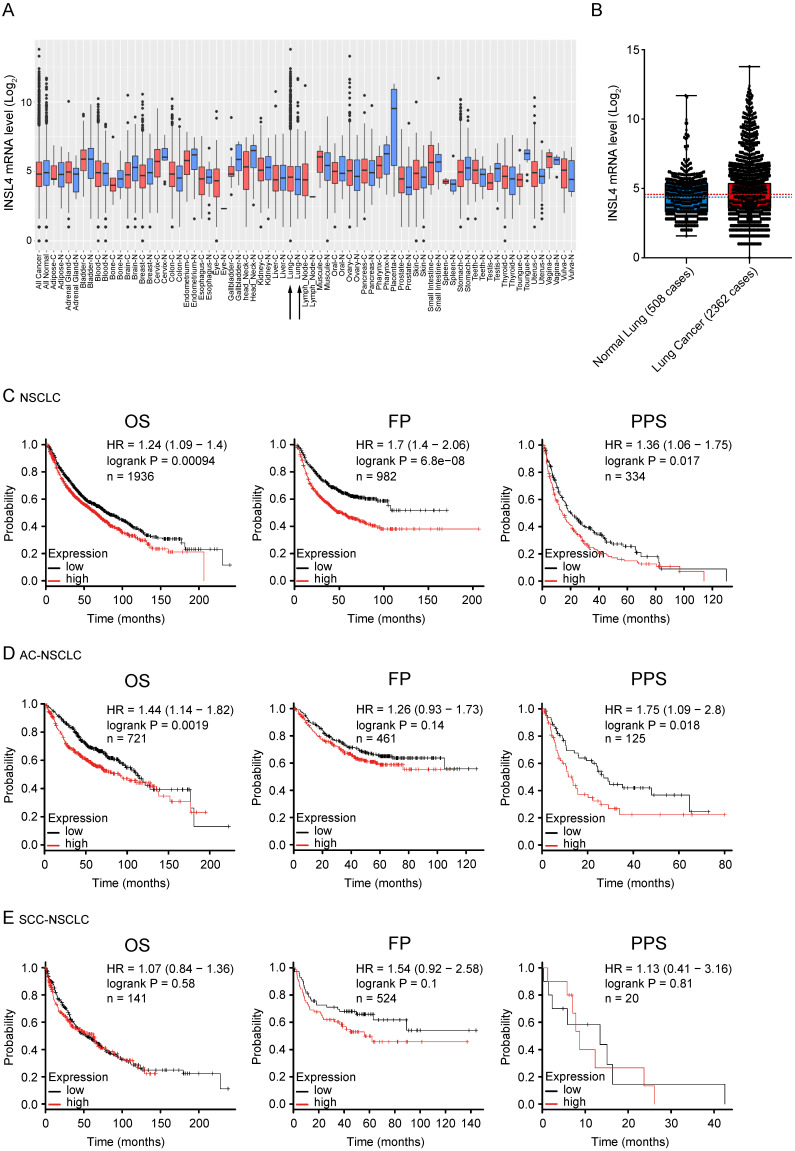
** INSL4 mRNA expression in patients with tumour and its prognostic value. A,** Extended INSL4 mRNA expression pattern across normal and cancer tissues was assessed using the GENT2 database. Arrow indicates lung tissue (Lung-C: lung cancer; Lung-N: normal lung). **B,** INSL4 expression pattern across normal lung and NSCLC tissues in U133Plus2.0 data set. A-B, refer to the 75th percentile, median and 25th percentile; dots refer to individual values. **C-E,** Kaplan-Meier plots for the whole cohort of NSCLC patients. The graphs plots were obtained using Kaplan-Meier -plot database and illustrate the prognostic effect of high and low expression of INSL4 in patients with lung cancer. OS= Overall Survival; FP= First Progression, PPS= Post Progression Survival (C) in NSCLC: OS (n=1936, P=0.00094, HR=1.24), FP (n=982, P=6.8e-08, HR= 1.7), and PPS (n=344, P=0.017, HR=1.36). **D,** in AC-NSCLC: OS (n=721, P=0.0019, HR=1.44), FP (n=461, P=0.14, HR= 1.26), and PPS (n=125, P=0.018, HR=1.75). **E,** in squamous carcinoma-NSCLC (SCC-NSCLC): OS (n=141, P=0.01, HR=1.54), FP (n=524, P=0.58, HR= 1.07), and PPS (n=20, P=0.81, HR=1.13). n= patient cohort.

As previously described *INSL4* is expressed in adult life in placenta and is expressed again in some tumours. To substantiate a clinically relevant tumourigenic role for INSL4, we used cancer outlier profile analysis as applied to the web-accessible GENT2 microarray database, containing samples from the Affymetrix U133Plus 2.0 platform. *INSL4* expression assessed in a set of different cancer histotypes revealed remarkable *INSL4* expression mostly occurring in LC tissues relative to control counterparts (Figure 6A and 6B). By extrapolating the analysis of a cohort of 2362 NSCLC patients and 508 normal lung samples, we observed a large prevalence of patients with *INSL4* overexpression (Figure 6B). Screening of the COSMIC data bank to evaluate *INSL4* overexpression in LC patients indicates that *INSL4* is overexpressed in almost 4% of all screened NSCLC patients. These results were in agreement with the findings above and reinforced the notion that *INSL4* is a potential oncogene 25.

To elucidate the association of *INSL4* expression with clinical endpoints in NSCLC patients, we used the Kaplan Meier Plotter. In all NSCLC patients, high *INSL4* expression was significantly associated with shortened Overall Survival (OS, *P*=0.00094, HR=1.24) as well as with reduced First Progression (FP, *P*=6.8e-08, HR=1.7) and Post Progression Survival (PPS, *P*=0.017, HR=1.36) (Figure 6C). Of note, in patients with AC-NSCLC, increased *INSL4* expression was significantly associated with poorer OS (*P*=0.0019, HR=1.44). A similar correlation was observed between *INSL4* status and PPS in AC-NSCLC patients (*P*=0.0018, HR=1.75) (Figure 6D). In contrast, there occurred no significant association between *INSL4* expression and clinical outcomes in patients with the squamous cell carcinoma-NSCLC (Figure 6E). Therefore, *INSL4* appears to be a specific marker of poor prognosis in patients with AC-NSCLC. These data confirm an important role played by *INSL4* in LC, but a selection in each patient has to be performed to discriminate the mRNA expression and the protein content to carry out specific therapy.

## Discussion

LC is a heterogeneous and dynamic disease associated with numerous somatic mutations, amplification and deletion in the cell's genome. NSCLC is the most common LC and is responsible for most cancer-related death worldwide 1, 5, 26, 27. Due to the poor clinical outcome, extensive studies have been aiming at clarifying the molecular mechanism involved in NSCLC onset, progression and recurrence, so to identify potentially druggable molecular targets and genes that could be exploited in prediction models for assessing the risk of recurrence. Molecular profile prediction of cancer is, indeed, key to the implementation of personalized therapeutic manoeuvres 7, 28, 29. Molecular targeted therapies are now being included in treatment regimens for LC patients as they have been shown to extend progression-free survival and improve overall survival 9, 30, 31.

INSL4 was first discovered in the placenta tissue and belongs in the relaxin/insulin-like family of peptides, which have been credited over the years with a functional role in cancer 12, 18, 21, 32-34. The functional consequences of relaxin receptor activation in cancer cells include increased cell motility 21 and tumour growth and angiogenesis 32, 35, all of which contribute to tumour expansion, tissue invasion and metastasis formation.

Here we demonstrate that *INSL4* is an active tumour-promoting gene in NSCLC, in that it favours proliferation, invasion and migration of LC cells. INSL4 overexpression increased mitosis in NSCLC H1299 cells and promoted cell-cycle progression. The biological relevance of INSL4 was substantiated in assays of colony-formation ability and migration. In particular, we observed that overexpression of INSL4 provides cells with an ability to generate colonies with higher cellular density, suggestive of loss of cell-contact inhibition and increased proliferation. On assaying INSL4-overexpressing cells in soft agar, we found that INSL4 affects the ability of transformed cells to acquire anchorage-independent growth 36, 37. Of note, INSL4 overexpression will also affect the original morphology and cytoskeleton arrangements in NSCLC H1299 cells, resulting in the appearance of large and numerous protrusions.

We also demonstrated that INSL4 autocrine and/or paracrine effects involve the activation and/or enhancement of the MAPK and AKT signalling pathways. Genetic mutations or Gain-Of-Function events can deregulate or hyperactivate the MAPK and/or AKT pathways during induction and progression of tumorigenesis. In particular, their activation is known to be key to LC development 22, 23, 38, 39. INSL4 could thus promote cell proliferation and invasiveness, by upregulating the MAPK and AKT signalling pathways, respectively. AKT activation is recognized as an important factor contributing to invasiveness of cancer cells 39, 40. Several studies have reported a crosstalk between the MAPK and AKT signalling pathways in order to sustain tumour cell proliferation 22, 23, 40.

The results from xenograft mouse model corroborated the *in vitro* data, further substantiating a role for INSL4 overexpression in tumour growth and invasiveness *in vivo.* Perhaps more importantly, the experimental data found validation in the analysis of patients with NSCLC. Cancer outlier profile analysis using the web-accessible database confirmed the tumour-promoting function of INSL4 as related to clinical outcome in NSCLC. We found high-level expression of *INSL4* in a majority of NSCLC surgical specimens.

However, important data have been achieved regarding the high discrepancy that we noted between *INSL4 gene* expression and protein content. Notably, we found that a direct correlation not always is present analysing *INSL4-mRNA* and protein. These results in cell lines and in NSCLC patients, outlines the importance to verify the protein content after gene expression profile. A previous paper describing the INSL4 role in proliferation showed, in LKB-1 mutated cells and patients, that a strict correlation is present between LKB-1 and *INSL4-mRNA* expression 41. Because the similar results have been obtained in protein stability in highly expressed INSL4 A549 and in low-expressed H1299 cell lines, we assert that the *INSL4-mRNA* is controlled at post-transcription level. Nevertheless, because *INSL4* is not expressed in normal lung, we observed that overexpression of *INSL4* significantly correlated with poor overall survival and post tumour progression survival in a large public clinical microarray database of 4,142 LC patients. In particular, elevated expression of *INSL4* correlated significantly with poor overall survival in a cohort of 721 AC-NSCLC patients. There was no correlation between *INSL4* expression and clinical outcomes in patients with squamous cell carcinomas of NSCLC. This observation strongly suggests that *INSL4* might serve as a specific predictive marker for AC-NSCLC, even if an accurate epigenetic analysis is required to ensure and provide a precision treatment to specific patient.

Overall, our study provides novel insight into INSL4 characterization and function in LC, and it establishes *INSL4* as a negative prognostic factor in AC-NSCLC. Moreover, the current study indicates that finding high level of mRNA not always correspond to elevated INSL4 protein content and a more accurate epigenetic analysis is required to evaluate *INSL4* as prognostic factor. Certainly, selected patients with elevated INSL4 protein could take advantage to inhibit *INSL4* and it may be an important strategy for alternative or combinatorial therapy in NSCLC patients. Finally, the results of this study could therefore contribute to an improved understanding of the molecular mechanisms involved in NSCLC carcinogenesis and likewise identifying new potentially druggable molecular targets in AC-NSCLC.

## Supplementary Material

Supplementary figures.

## Abbreviations

INSL4: insulin-like 4
NSCLC: non-small cell lung cancer
LC: lung cancer
AD-NSCLC: adenocarcinoma-non-small cell lung cancer
MAPK: mitogen-activated protein kinase
MEK: mitogen-activated protein kinase kinase
AKT: protein-serine-threonine kinase
ER: early relapse
NR: no relapse
RLN/INSL: relaxin/insulin-like
RXFP: RelaXin family peptide
OS: overall survival
FP: first progression
PPS: post progression survival
SPP: signal-peptide peptidase
IFS: insulin family signature
VEGFR: vascular endothelial growth factor
