# Supplementary material for: INSL4 as prognostic marker for proliferation and invasiveness in Non-Small-Cell Lung Cancer
Source: J Cancer. 2021 May 5;12(13):3781–95. doi: 10.7150/jca.51332 (PMC8176261; doi:10.7150/jca.51332)
Supplement: Supplementary file 1 — Supplementary figures. [file jcav12p3781s1.pdf]

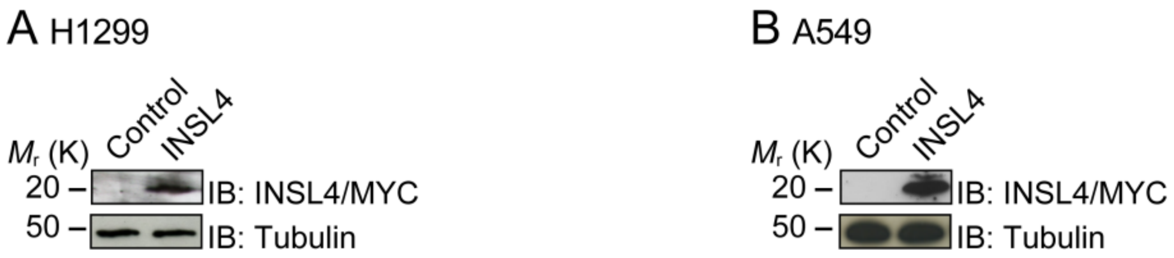

Supplementary Figure S1. INSL4 transfection in H1299 and A549 cell lines. A-B, Transfection efficiency of the fusion protein INSL4-Myc-Tag in (A) H1299 and in (B) A549 cell lines was evaluated by immunoblot analysis using anti Myc-Tag. Anti-Tubulin is used as loading control.

A Placenta

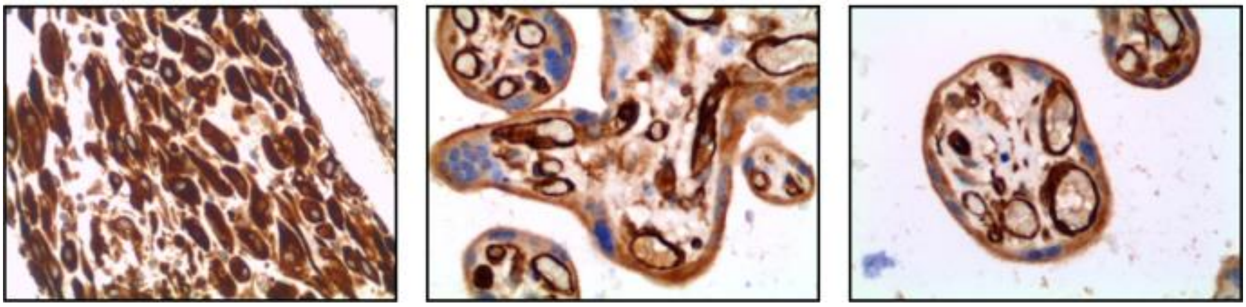

Supplementary Figure S2. A, INSL4 immunohistochemistry staining for assessing a positive control for anti-INSL4 antibody using human placenta slides. In pictures placental cytotrophoblast and syncytiotrophoblast are showed. Magnification x400.
